# Systemic delivery of anti‐sense oligonucleotide targeting α‐synuclein for the treatment of multiple systems atrophy

**DOI:** 10.1002/alz70855_105773

**Published:** 2025-12-24

**Authors:** Robert A. Rissman, Brian Spencer

**Affiliations:** ^1^ Alzheimer's Therapeutic Research Institute, University of Southern California, San Diego, CA, USA; ^2^ Alzheimer's Therapeutic Research Institute, Keck School of Medicine, University of Southern California, San Diego, CA, USA

## Abstract

**Background:**

Multiple Systems Atrophy (MSA) is a rare, sporadic, age‐related synucleinopathy characterized by Parkinson‐like motor symptoms and ataxia. There is no therapy for MSA other than symptomatic treatment. MSA is characterized pathologically by glial cytoplasmic inclusions (GCI) of α‐synuclein (αSyn) occurring in oligodendrocytes leading to loss of myelination in the brain. We recently utilized a peptide‐mediated delivery method to systemically transport an anti‐sense oligonucleotide (ASO) targeted to αSyn in a mouse model of MSA.

**Method:**

We hypothesized that systemic delivery of αSyn ASO by peptide mediated delivery to a mouse model of MSA would reduce the αSyn accumulation in oligodendrocytes and reduce the overt pathology associated with MSA.

**Result:**

Following monthly treatments of the αSyn ASO, we found increased myelination in the corpus callosum and the cerebellum. We also observed increased numbers of oligodendrocytes and reduced gliosis; however, we did not detect changes in overall αSyn in the areas of the brain we examined. Upon further analysis, we determined the peptide‐mediated delivery of αSyn ASO was not taken up by oligodendrocytes.

**Conclusion:**

Thus, we have successfully alleviated some of the pathology associated with MSA in a mouse model; however, without direct delivery to oligodendrocytes, other approaches may need to supplement this therapy.

